# Low Temperature HCHO Detection by SnO_2_/TiO_2_@Au and SnO_2_/TiO_2_@Pt: Understanding by In-Situ DRIFT Spectroscopy

**DOI:** 10.3390/nano11082049

**Published:** 2021-08-11

**Authors:** Abulkosim Nasriddinov, Vadim Platonov, Alexey Garshev, Marina Rumyantseva

**Affiliations:** 1 Department of Chemistry, Lomonosov Moscow State University, Leninskie Gory 1-3, 119991 Moscow, Russia; a.f.nasriddinov@gmail.com (A.N.); agnes1992@yandex.ru (V.P.); gaal@mail.ru (A.G.); 2 Department of Materials Science, Lomonosov Moscow State University, Leninskie Gory 1-3, 119991 Moscow, Russia

**Keywords:** nanocrystalline SnO_2_, SnO_2_/TiO_2_ nanocomposite, Au and Pt modification, formaldehyde gas sensor, DRIFTS investigation

## Abstract

In this work we analyze the effectiveness of decoration of nanocrystalline SnO_2_/TiO_2_ composites with gold nanoparticles (Au NPs) and platinum nanoparticles (Pt NPs) in enhancing gas sensor properties in low-temperature HCHO detection. Nanocrystalline SnO_2_/TiO_2_ composites were synthesized by a chemical precipitation method with following modification with Pt and Au NPs by the impregnation method. The nanocomposites were characterized by TEM, XRD, Raman and FTIR spectroscopy, DRIFTS, XPS, TPR-H_2_ methods. In HCHO detection, the modification of SnO_2_ with TiO_2_ leads to a shift in the optimal temperature from 150 to 100 °C. Further modification of SnO_2_/TiO_2_ nanocomposites with Au NPs increases the sensor signal at T = 100 °C, while modification with Pt NPs gives rise to the appearance of sensor responses at T = 25 °C and 50 °C. At 200 °C nanocomposites exhibited high selectivity toward formaldehyde within the sub-ppm concentration range among different VOCs. The influence of Pt and Au NPs on surface reactivity of SnO_2_/TiO_2_ composite and enhancement of the sensor response toward HCHO was studied by DRIFT spectroscopy and explained by the chemical and electronic sensitization mechanisms.

## 1. Introduction

In recent years, gas sensors for detecting formaldehyde (HCHO) have received increasing attention because of the harmful impacts of HCHO on human health [1,2]. Formaldehyde is one of the most common sources of indoor air pollution, as it is widely used in various coatings for building materials and furniture [3]. Long-term exposure to HCHO leads to chronic and incurable diseases. As a harmful substance, formaldehyde can cause serious health damage even at low concentrations. Therefore, the main challenge in HCHO detection is necessity to determine low target concentrations. According to the WHO requirements, the corresponding threshold in indoor working area is 0.5 mg/m^3^ (0.4 ppm) [1].

Effectively detecting toxic gases, including formaldehyde, requires a sensitive, selective and easy-to-design analytical sensor. Conductometric gas sensors based on metal oxide semiconductors (MOS) are among the most promising devices. SnO_2_ is a well-proven sensing material that has a high sensitivity to various gases, including HCHO [4,5,6].

The use of the nanocomposites with *n-n* and *p-n* MOS heterocontacts, in particular SnO_2_/ZnO [7,8], SnO_2_/In_2_O_3_ [9], SnO_2_/TiO_2_ [10,11,12,13,14], SnO_2_/Fe_2_O_3_ [15], SnO_2_/NiO [16,17,18,19] and SnO_2_/carbon-based materials [20,21] in comparison to pure oxides, can significantly enhance the sensor response toward formaldehyde and other volatile organic compounds (VOCs) and reduce the operating temperature. The main idea is based on controlling the amount of charge carriers at the interface that, in turn, provides an increase in the concentration of chemisorbed oxygen participating in the oxidation reactions that cause a sensor response. Furthermore, SnO_2_/TiO_2_ based composites demonstrated significant efficiency in catalytic oxidation of VOCs and photocatalytic degradation of organic compounds due to additional high catalytic activity of TiO_2_ phases [11,22,23,24].

One of the promising approaches for improving and controlling MOS selectivity and sensitivity is surface decoration [22,25]. Currently, well-dispersed noble metal nanoparticles are predominantly used as catalytic modifiers, since they facilitate the oxidation of reducing gases, in particular VOCs, on the semiconductor surface due to a decrease in the activation energy of the oxidation reaction [22,26,27,28]. By doing this, they can significantly reduce the operating temperature of gas detection down to room temperature [29,30]. These additives can be located on the surface of semiconductor oxides in the form of clusters or individual nanoparticles of various sizes [31]. The influence of catalytic modifiers on the formation of a sensor signal of semiconductor oxides can be realized through chemical and electronic mechanisms [25,32]. From this point of view, the study of surface reactivity along with gas sensor properties will allow the finding of obscure issues, and obtaining of a key to further development and improvement of MOS gas sensors.

Au and Pt NPs have an exceptional role in the formation of oxygen vacancies in solid oxides that enhance their activity in reducing gas oxidation [33]. There are several works describing the effectiveness of Au incorporation into mixed metal oxide nanomaterials in oxidation reactions at a temperature close to 100 °C. Yang and Li [34] noticed that the introduction of Au nanoparticles leads to a significant increase in the catalytic conversion of CO on the surface of Co_3_O_4_/CeO_2_ heterostructures. The process begins at T = 100 °C, and the complete CO conversion is observed at T = 127 °C. The same effect was observed by Li et al. [35]: the presence of Au nanoparticles strongly contributed to the reduction of CeO_2_ in Au/CeO_2_–TiO_2_ nanorods, increasing the redox ability and catalytic activity of the material that led to a complete CO conversion at T = 120 °C. In the review [36] it was shown that the decoration of metal oxides with Au NPs with a particle size of ~20 nm can increase the catalytic activity and sensor response toward CO and ethanol and shift the operating temperature to the 100–150 °C range and even lower. In addition, as reported by Marikutsa et al. [26] TiO_2_/Au sensors demonstrated the highest sensitivity to VOCs among different Au-functionalized *n*-type and *p*-type metal oxide semiconductors. They attributed this result to a combination of the catalytic effect of gold and the proper Ti–O binding energy, which is favorable for the chemisorption of oxygen and its release when interacting with VOCs molecules.

In the present work, we have focused our attention on the effect of Au and Pt nanoparticles (NPs) as surface catalytic modifiers on sensing mechanisms of the SnO_2_/TiO_2_ composite in low temperature HCHO detection. Experimental results showed that modification with Au and Pt NPs leads to a shift of the favorable operating temperature to the low temperature range and at the same time amplifies the sensor signal.

## 2. Materials and Methods

### 2.1. Materials Synthesis

#### 2.1.1. Synthesis of Au Sol

Colloidal gold nanoparticles (Au NPs) were obtained by the well-known technique [37]. Briefly, 2.0 mL of a 1% sodium citrate solution Na_3_C_6_H_5_O_7_ (>99%, Sigma-Aldrich, St. Louis, MO, USA) was quickly added to 20 mL boiling solution of 1 mM HAuCl_4_·3H_2_O (Sigma-Aldrich) with vigorous stirring. The solution was boiled until the color became ruby red and then cooled to room temperature (RT).

#### 2.1.2. Synthesis of Nanocomposites

At the first stage (Figure S1, Supplementary Materials) SnO_2_ × H_2_O gel was precipitated from H_2_SnCl_6_ (Sigma-Aldrich, St. Louis, MO, USA) solution by dropwise adding of aqueous ammonia (25%) until pH ~ 7. After washing, drying (T = 80 °C, 24 h) and annealing (T = 300 °C 24 h) nanocrystalline SnO_2_ was obtained. At the next step, H_2_TiCl_6_ solution (Component-reactive, Russia) was added to the SnO_2_ aqueous suspension under intensive stirring, and then aqueous ammonia (25%) was added dropwise. The gel-like precipitate was washed, dried and annealed in air at 300 °C for 24 h to obtain the SnO_2_/TiO_2_ composite. The resulting solid phase at the last stage was impregnated with Pt(acac)_2_ (Sigma-Aldrich, Buchs, Switzerland) ethanol solution (1.5 mM) or previously formed Au sol (1 mM) and then annealed at 300 °C for 24 h. The amount of the introduced modifier was selected so that the ratio of Pt/Sn or Au/Sn was 1.0 wt.%.

### 2.2. Materials Characterization

The size and shape of the Au NPs were analyzed by LEO 912AB Omega transmission electron microscope (TEM) (Carl Zeiss, Germany). The morphology and particle size analysis, selected area electron diffraction (SAED) images, and scanning transmission electron microscopy in high-angle annular dark-field mode (STEM-HAADF) images of the nanocomposites were registered on Libra 200 TEM (Carl Zeiss, Germany) with a cathode with thermal field emission at an accelerating voltage of 200 kV. The images were obtained using an Ultra Scan 4000 CCD camera (Gatan Inc., Las Positas Blvd. Pleasanton, CA, USA). The energy-dispersive X-ray spectroscopy (EDX) signal was recorded on a silicon drift X-MAX 80 T detector (Oxford Instruments, Abingdon, Oxfordshire, England). The images were processed using the ImageJ software (NIH).

X-ray diffraction (XRD) patterns were collected on a DRON-4 diffractometer using monochromatic Cu Kα radiation in the 2θ range of 10–80° with a 0.1° step. SnO_2_ crystallite size (d_XRD_) was calculated from the broadening of the most intensive XRD peaks using Scherrer Equation (1).

(1) $$d=\frac{k*\lambda }{\beta *cos\Theta }$$

where *d* is the mean crystallite size (nm), *k* is a dimensionless shape factor and is about 0.9, λ = 1.5406 Å is the X-ray wavelength, *β* is the line broadening at the half of maximum intensity, and *Θ* is the Bragg angle. The phase composition was determined using the WinXPOW software.

The specific surface area was measured by nitrogen adsorption with the Chemisorb 2750 instrument (Micromeritics, Norcross, GA, USA). The chemical composition of the samples was analyzed using M1 Mistral X-ray fluorescent (XRF) spectrometer (Bruker, Billerica, MA, USA).

Raman spectra were recorded on an i-Raman Plus spectrometer (BW Tek, 19 Shea Way, Newark, NJ, USA) equipped with a BAC 151C microscope in the range of 90–1000 cm^−1^ with a resolution of 4 cm^−1^. A green laser (532 nm) was used as a radiation source.

The absorption spectrum of a sol of Au nanoparticles stabilized with sodium citrate was obtained on a Cary 50 (Varian Inc., Melbourne, Australia) spectrophotometer. The survey was carried out in the 200–800 nm range; the baseline of deionized water was subtracted.

A Perkin Elmer Frontier spectrometer (Perkin Elmer Inc., Beaconsfield, UK) was used to register Fourier-transformed infrared (FTIR) and diffuse-reflectance infrared Fourier-transformed (DRIFT) spectra. The FTIR spectra were registered in the region of 4000–400 cm^−1^ with a step of 1 cm^–1^. The FTIR measurements were carried out in the transmission mode; 0.3–0.5 mg of the samples were ground with 40 mg of KBr (FT-IR grade, Sigma-Aldrich) and pressed into tablets ~0.5 mm thick and 12 mm in diameter. The baseline was preliminarily taken from pure KBr. The DRIFT spectra were obtained both at RT and at T = 100 °C using diffuse reflectance accessory DiffusIR annex and heated flow chamber HC900 (Pike Technologies, Cottonwood Dr., Madison, WI, USA) sealed by KBr window. The samples (50 mg) were placed in sapphire crucibles (5 mm diameter) and pre-annealed at T = 300 °C in a flow of pure air (1 h) to clean the surface from the contaminations. A gas mixture containing 20 ppm of formaldehyde in dry air was used for the investigations.

Thermo-programmed hydrogen reduction was carried out on a Chemisorb 2750 (Micromeritics, Norcross, GA, USA) device in a quartz reactor at a gas flow of 10% H_2_ in argon (50 mL/min) at a heating rate of 10 °C/min from 25 °C to 900 °C.

The chemical state of the elements was analyzed by X-ray photoelectron spectroscopy (XPS) on Omicron ESCA+ (Germany) with a monochromatic aluminum anode (AlK_α_, E = 1486.6 eV) using a neutralizer (scanning step 0.1 eV/s, transmission energy 20 eV). The spectra were processed using the UNIFIT software package. The peaks were approximated by convolution of the Gauss and Lorentz functions with the simultaneous optimization of the background parameters.

### 2.3. Sensor Fabrication and Gas Sensing Measurements

The electrical conductivity of the samples in a gas flow was measured on specially designed micro-hotplates with platinum electrodes on the front side and a platinum micro-heater on the back side. The suspensions of nanocomposite powders in α-terpineol (Sigma-Aldrich) were drop-coated on the micro-hotplate substrates (dielectric Al_2_O_3_, area: 0.9 mm × 0.9 mm, thickness 0.15 mm). Thick films were annealed at 300 °C for 5 h to remove the binder and sinter the particles. Gas sensor properties were studied in-situ in the temperature range of 25–300 °C in a flow cell under a controlled gas flow of 100 ± 0.1 mL/min. The gas mixtures containing 0.12–0.25–0.5–1.0 ppm HCHO were obtained by dilution of the gas from an attested gas mixture (35 ppm of HCHO in N_2_) with background purified air. The measurements were carried out with a periodic change in the gas phase composition (15 min target gas/15 min air).

## 3. Results and Discussion

### 3.1. Morphology and Composition Characterization

**Figure 1:**
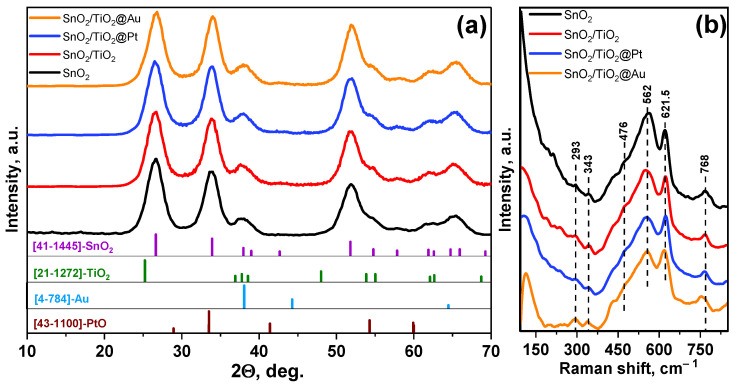
XRD patterns (**a**) and Raman spectra (**b**) of nanocomposites.

The phase composition of the obtained samples (Figure 1a) was characterized using the structural parameters from the ICDD PDF-2 database of SnO_2_ cassiterite (41–1445), TiO_2_ anatase (21–1272) (Figure S2, Supplementary Materials), Au (4-784) (Figure S3, Supplementary Materials) and PtO (43–1100). The XRD patterns clearly show the reflections corresponding to the SnO_2_ phase with the tetragonal cassiterite structure. Other possible crystalline phases are not detected, since their concentration is low, and their diffraction maxima overlap with the intense reflections of the SnO_2_ phase.

Figure 1b represents Raman spectra of the synthesized samples. All spectra have almost the same peaks at the same positions, which mean no phase transition during synthesis process. The peaks detected at 476, 621.5 and 768 cm^−1^ correspond to the SnO_2_ E_g_, A_1g_ and B_2g_ fundamental vibration modes, respectively. The intense and wide band at 562 cm^−1^ is due to the surface mode of the nano-sized SnO_2_ [38,39]. A decrease in the intensity of the SnO_2_ surface mode in the spectra of nanocomposites indicates that the modifiers are localized on the surface of SnO_2_ grains. The peaks at 293 and 343 cm^−1^ are related to transformation of IR to Raman active modes [38].

**Figure 2:**
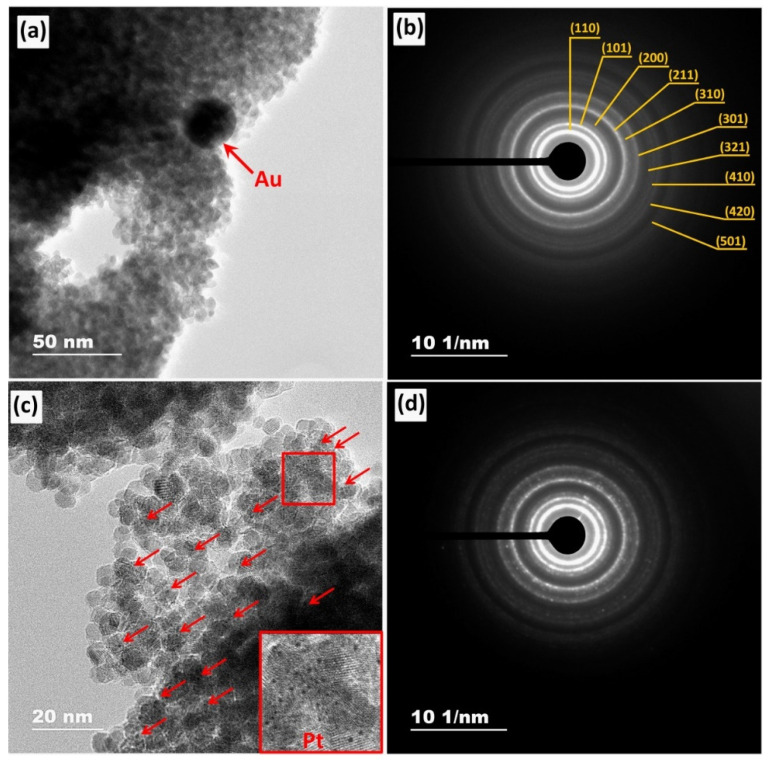
TEM images and their corresponding SAED patterns of SnO_2_/TiO_2_@Au (**a**) and (**b**) and SnO_2_/TiO_2_@Pt (**c**) and (**d**) nanocomposites. Pt nanoparticles are marked with red arrows (**c**).

The transmission electron micrographs and corresponding selected-area electron diffraction (SAED) patterns of SnO_2_/TiO_2_@Au and SnO_2_/TiO_2_@Pt nanocomposites are shown in Figure 2a,b and Figure 2c,d, respectively.

According to TEM images and interplanar spacing analysis, the samples are well crystallized and consist of near spherical SnO_2_ crystallites with an average size of 4.5 ± 1 nm that is similar to those calculated from Scherrer’s equation (Figure S4, Supplementary Materials). The Au NP (Figure 2a) has a spherical shape with the mean diameter of 17 ± 4 nm (Figure S3, Supplementary Materials). The small black dots that can be observed in Figure 2c (indicated by red arrows), are probably related to platinum-containing nanoparticles. The SAED patterns (Figure 2b,d) consist of concentric rings, which are typical for nanocrystalline samples. The measured values of interplanar spacing (d_hkl_) correspond to the rutile crystalline structure of SnO_2_ (ICDD, No. 41–1445). The reflections for TiO_2_ anatase phase, Au and Pt NPs, are not clearly seen due to the same reasons as in the case of XRD.

**Figure 3:**
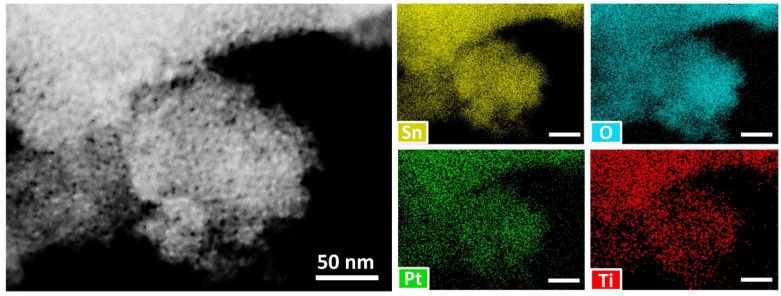
HAADF-STEM image and corresponding EDX mapping of the Sn, O, Pt, Ti signals of the SnO_2_/TiO_2_@Pt nanocomposite.

The presence of Pt NPs on the surface of SnO_2_/TiO_2_ nanocomposite was additionally proved by HAADF-STEM with EDX mapping (Figure 3) and elemental analysis. According to these results the Pt NPs and Ti-containing phase are finely dispersed on the surface of SnO_2_ grains; the concentration of modifiers determined by XRF analysis is approximately equal to the preassigned one (Table 1).

### 3.2. Characterization of Surface Composition and Chemical State

**Figure 4:**
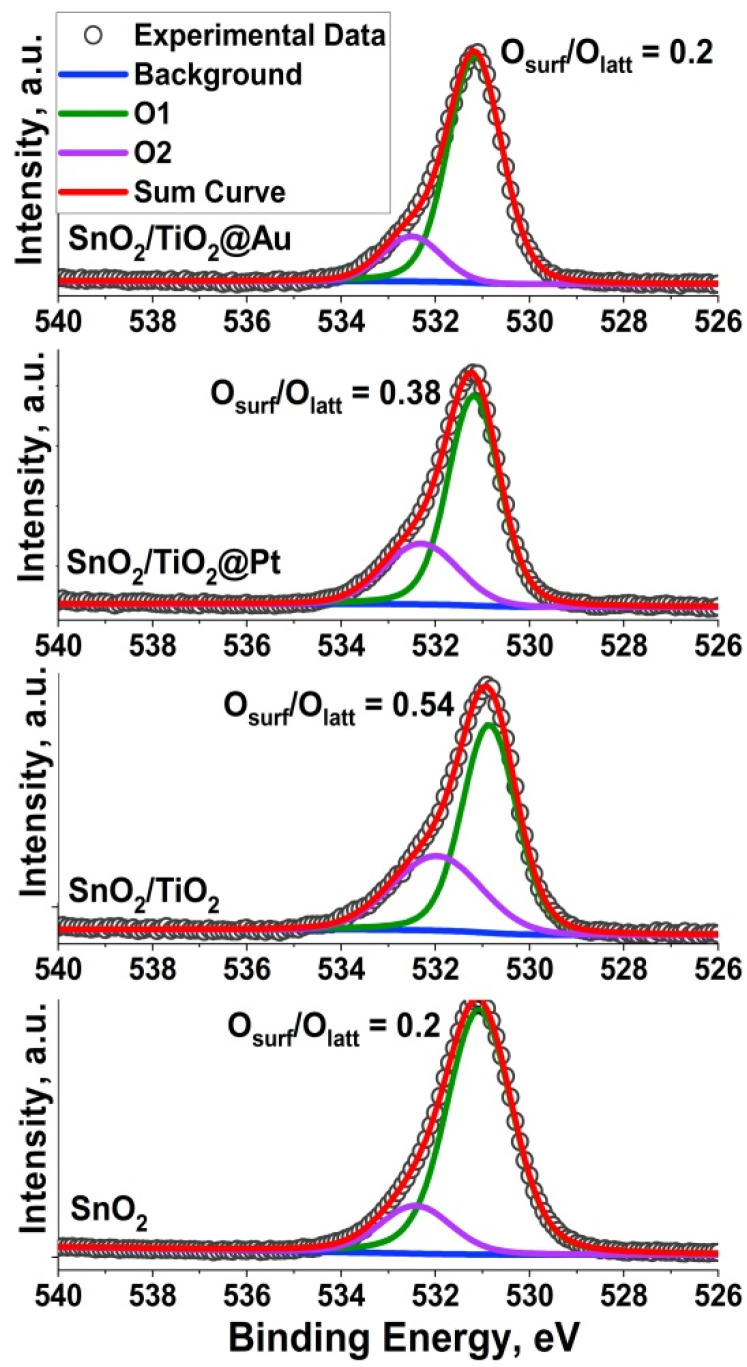
O1s XP-spectra of the synthesized samples.

The surface composition and chemical state of the elements were investigated by the XPS method. Sn 3d XP-spectra are shown in Figure S5 (Supplementary Materials). In all samples, tin presents as Sn (IV); the peaks at 487.2 and 495.6 eV were assigned to Sn 3d_5/2_ and Sn 3d_3/2_, respectively [40]. Figure 4 describes the O1s spectra of all samples. Two components in the O1s spectra locating at 531.1 eV and 532.4 eV are attributed to lattice oxygen (O_lat_) and chemisorbed (O_surf_) oxygen species/hydroxyl ions (O^−^, O^2−^ and OH^−^), respectively. Compared to SnO_2_, the ration O_surf_/O_lat_ for the SnO_2_/TiO_2_ sample is 2 times greater and indicates the presence of more chemisorbed surface oxygen species after synthesis. The formation of *n-n* heterocontact at the SnO_2_/TiO_2_ interface can lead to electron transfer from the TiO_2_ conduction band into the SnO_2_ conduction band. A high concentration of electrons, in turn, can lead to increased chemisorption of oxygen species [10].

**Figure 5:**
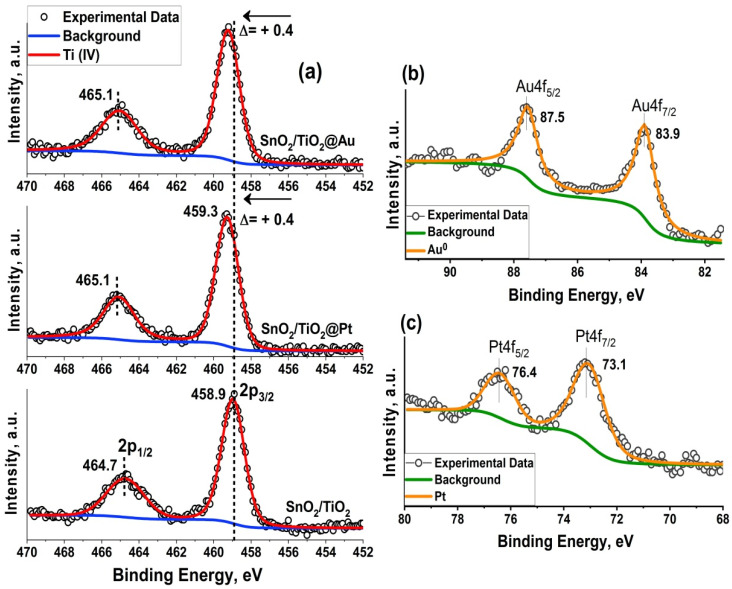
Ti 2p (**a**), Au 4f (**b**) and Pt 4f (**c**) XP-spectra of the synthesized samples.

Ti 2p XP-spectra are shown in Figure 5a. The peaks with binding energies of 458.9 and 464.7 eV are ascribed to the Ti 2p_3/2_ and 2p_1/2_ peaks of Ti(IV), respectively [40]. The binding energy of Ti 2p peak is shifted positively by 0.4 eV both for SnO_2_/TiO_2_@Pt and SnO_2_/TiO_2_@Au samples. It could be assigned to the electron transfer from TiO_2_ to Au and Pt NPs, respectively, due to the difference of work function and metal-support interactions. [41,42]. The observed situation can also be the reason for the decrease in the O_surf_/O_lat_ ratio for the SnO_2_/TiO_2_@Pt and SnO_2_/TiO_2_@Au samples (Figure 4).

Figure 5b,c shows the deconvoluted Pt 4f and Au 4f spectra, respectively. According to [40,42,43], the peaks located at 73.1 eV (Pt 4f_7/2_) and 76.4 eV (Pt 4f_5/2_) may be associated with Pt (II) in PtO or Pt(OH)_2_. However, the FTIR spectra (Figure S6, Supplementary Materials) showed that the amount of the hydroxyl groups is almost similar in all samples. Therefore, we can conclude that platinum in the composite is in the oxidized form of PtO. The long-term (24 h) and high-temperature (300 °C) annealing could lead to oxidation of the surface layer of platinum nanoparticles. The Au 4f XP-spectrum indicates the presence of only metallic Au NPs [40].

**Figure 6:**
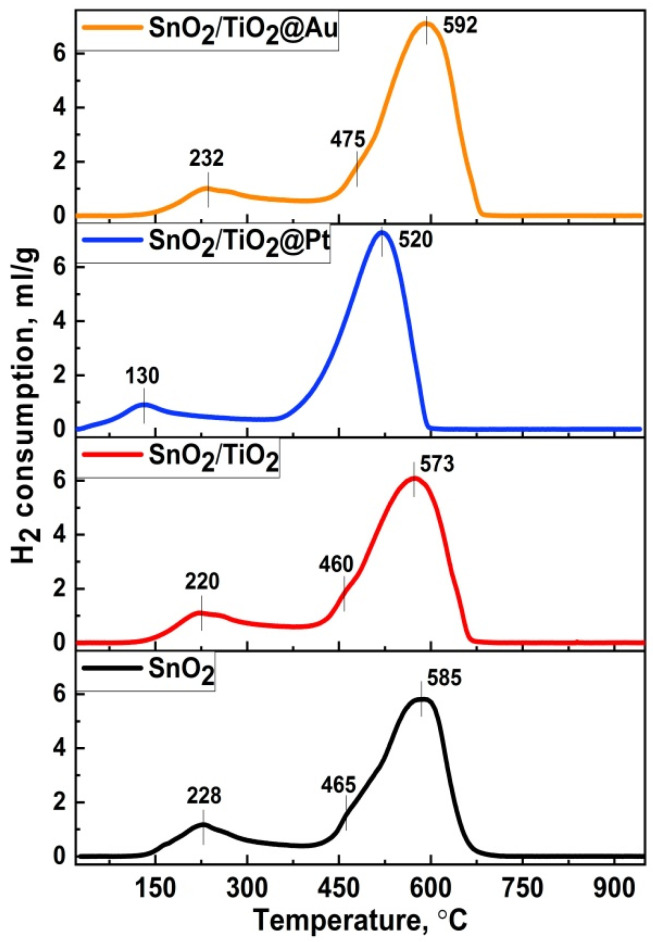
TPR-H_2_ spectra of the synthesized samples.

The method of temperature-programmed reduction of hydrogen (TPR-H_2_) was used to study the oxidative active centers on the surface of the samples. The TPR curves are shown in Figure 6. The profiles of hydrogen consumption can be divided into two temperature regions: the low-temperature one (T = 100–300 °C) corresponds to the reduction of the surface oxygen-containing species. The high-temperature region (T = 400–750 °C) corresponds to the reduction of SnO_2_ to metallic Sn. A small shoulder can be observed in the high-temperature region at T = 460–465 °C that corresponds to a partial reduction of SnO_2_ to SnO.

The introduction of modifiers has a significant effect on the shape and position of hydrogen consumption peaks both in the high-temperature and low-temperature regions. In particular, the introduction of TiO_2_ and Pt NPs leads to a noticeable shift of the hydrogen consumption peaks to the low-temperature region. This effect of decreasing the activation energy of oxide reduction is due to the high catalytic activity of modifiers that can lead to a spillover effect [44,45]. However, the introduction of gold nanoparticles, on the contrary, shifts H_2_ consumption peaks to the high-temperature region. This effect can be attributed [46,47] to the interaction of gold nanoparticles with surface oxygen vacancies on the SnO_2_ surface and, as a consequence, the localization of chemisorbed oxygen species at the triple phase interface “Au-SnO_2_-gas”. Fujita et al. [48] investigated the dependence of the effectiveness of CO conversion on M–O bond energy for Au/MO_x_ catalysts. They reported that catalytic activity of Au/TiO_2_ has the highest value among the large variety of metal oxides, which was proposed to be due to the formation of oxygen vacancies in the perimeter region between Au NPs and TiO_2_ contact. Green et al. have developed a model according to which an Au–Ti^4+^ dual site became a favorable adsorption site due to the O_2_ activation [49]. It was also found from DFT calculations [50] that at the low operating temperatures metallic Au NPs are more effective for CO oxidation then Au^δ+^ due to the low barrier (0.1 eV) of electron transfer from Au particles to chemisorbed oxygen located at the Au/TiO_2_ interface.

### 3.3. Gas Sensor Properties

**Figure 7:**
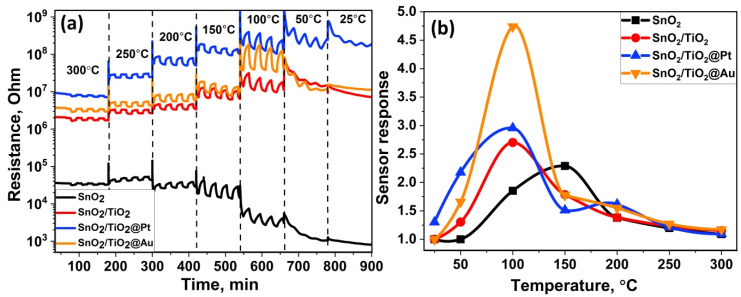
Change in resistance of the samples under periodic change of the gas phase composition in the temperature range of 300–25 °C (**a**); temperature dependencies of the sensor response of nanocomposites toward 1 ppm HCHO (**b**).

The sensor properties of the synthesized nanocomposites were investigated toward 1 ppm formaldehyde in the temperature range of 300–25 °C in order to determine the optimal operating temperature. Figure 7a shows a dynamic resistance change for nanocomposites during exposure of pure air (15 min)—HCHO (15 min). Sensors’ resistance reversibly decreases in a HCHO atmosphere and increases in pure air, which is characteristic behavior for *n*-type semiconductors. The sensor response of the samples toward HCHO was calculated by the following relation: ***S = R(air)/R(gas)***, where ***R(air)*** is a resistance in pure air, and ***R(gas)***—is a resistance in target gas. Typical bell-shaped plots of the temperature dependence of the sensor signals are shown in Figure 7b.

It was found that modification of SnO_2_ with titanium dioxide leads to a shift in the optimal temperature of sensor response from 150 to 100 °C. Modification of SnO_2_/TiO_2_ nanocomposites with Au NPs increases the sensor signal at T = 100 °C, while modification with Pt NPs gives rise to the appearance of sensor response at T = 25 °C and 50 °C.

However, the results of low temperature measurements (below 200 °C) show a significant baseline drift, which makes it impossible to use these conditions in practice. Therefore, the concentration dependences of the sensor signal were obtained at T = 200 °C, which provides a reproducible and stable resistance change depending on the composition of the gas phase.

**Figure 8:**
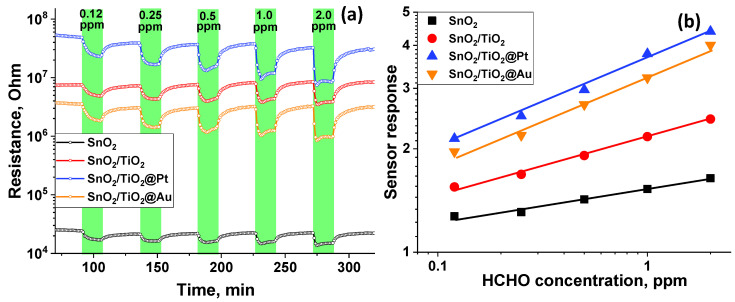
Change in the nanocomposites’ resistance depending on HCHO concentration of HCHO (**a**) and calibration curves (**b**) at T = 200 °C.

The calibration curves are linear in double logarithmic coordinates (Figure 8), which allows us to calculate the minimum detectable HCHO concentration ***c_min_***. The ***c_min_*** values (Table 2) were estimated from calibration curves using the ratio ***R(av)/(R(av)-3σ)*** as a minimum measurable sensor response, where ***R(av)*** is an average resistance in pure air, and ***σ*** is a standard deviation of resistance in pure air [51]. The Au modified sample demonstrated the lowest value of the minimum detectable HCHO concentration.

**Figure 9:**
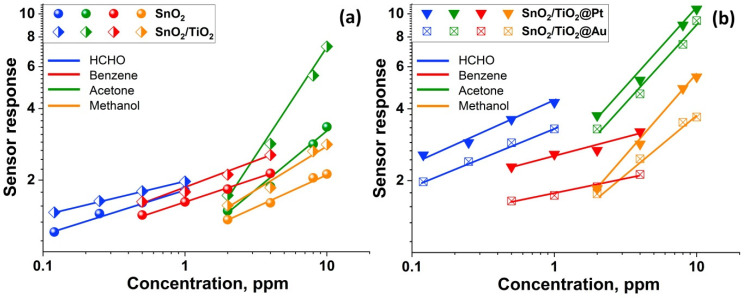
Cross sensitivity of SnO_2_, SnO_2_/TiO_2_ (**a**) and SnO_2_/TiO_2_@Pt, SnO_2_/TiO_2_@Au (**b**) samples toward HCHO (blue), benzene (red), acetone (green) and methanol (orange) at 200 °C.

The cross sensitivity of the samples was investigated in detection of different VOCs: formaldehyde, benzene, acetone and methanol at 200 °C (Figure 9). The concentration of VOCs was selected based on a corresponding threshold in an indoor working area [1,4,52]. It was observed that Pt and Au modified samples have enhanced sensor responses toward VOCs. It is obvious that in the sub-ppm concentration range, SnO_2_/TiO_2_ based sensors demonstrate high selective sensitivity when detecting formaldehyde.

### 3.4. Investigation of Surface Reactivity

**Figure 10:**
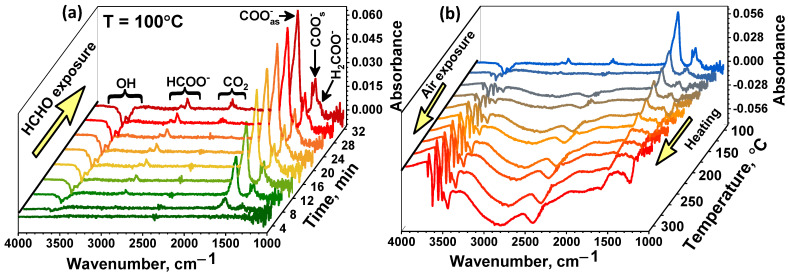
In situ DRIFT spectra of the SnO_2_/TiO_2_@Pt sample during HCHO adsorption at T = 100 °C (**a**) and during heating in air atmosphere after HCHO adsorption (**b**).

The surface reactivity of composite materials was investigated by the DRIFTS method. Figure 10a shows the change of the in situ DRIFT spectra of the SnO_2_/TiO_2_@Pt sample in the presence of 20 ppm HCHO at T = 100 °C. During HCHO exposure, the bands located at 1290 cm^−1^, 1345 cm^−1^, 1385 cm^−1^, 1560 cm^−1^, 1620 cm^−1^, 2340 cm^−1^, 2886 cm^−1^, and at 2968 cm^−1^ begin to increase, which indicates the accumulation of new functional groups on the MOS surface. The appearance of the bands at 1345 cm^−1^ and 1560 cm^−1^ were assumed to originate from the COO^-^ symmetric stretching and COO^-^ asymmetric stretching of formate species, respectively [53,54]. Absorption bands related to υ(CH) and υ_s_(OCO) of HCOO^-^ species are located at 2886 cm^−1^, 2968 cm^−1^ and 1385 cm^−1^, respectively [54,55]. The peak at 1290 cm^−1^ is due to the τ(CH_2_) vibration mode and indicates the appearance of dioxymethylene (DOM, H_2_COO^-^) intermediate [55].

The full oxidation of formate species over the nanocomposites was observed by appearance of the band at 2340 cm^−1^, which is characterized by the adsorbed CO_2_ molecules. The accumulation of surface H_2_O molecules can be evidenced by an increase in peak intensity at 1620 cm^−1^. Another group of active sites that can affect the oxidative activity of the materials are surface hydroxyl groups. The negative peaks associated with vibrational frequencies of free surface hydroxyls on oxides were observed at 3598 cm^−1^, 3670 cm^−1^ and 3724 cm^−1^ [56].

When purified air launched into the cell after the analyte gas was closed at T = 100 °C, desorption of formate groups does not occur completely, which causes a baseline drift observed in the sensor measurements (Figure 7a). Only heating up to 300 °C leads to the complete desorption of these groups (Figure 10b). A decrease in the value of the sensor signal with an increase in the measurement temperature to 250–300 °C is due to the contribution of thermal desorption of chemisorbed oxygen species, which play a key role in the oxidation of formaldehyde, as well as to desorption of formaldehyde molecules themselves.

**Figure 11:**
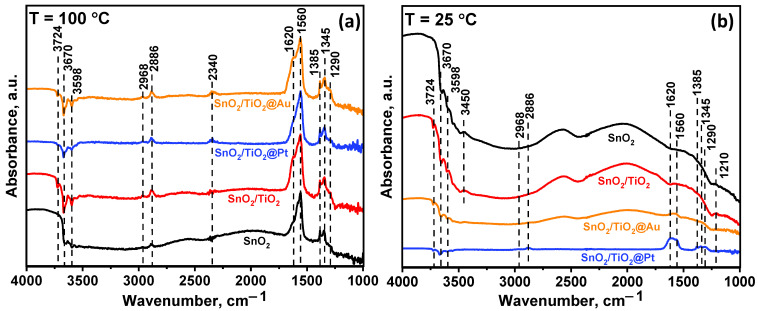
In situ DRIFT spectra of the samples after 40 min HCHO (20 ppm) exposure at T = 100 °C (**a**) and T = 25 °C (**b**).

The profile and peaks of DRIFT spectra of the samples at 100 °C indicate that the same intermediate products were formed during formaldehyde oxidation on the surface of all nanocomposites (Figure 11 a). At the same time, at RT, the appearance of bands of formate and DOM groups is observed only in the SnO_2_/TiO_2_@Pt sample (Figure 11b). The spectra of other samples show broad negative humps in hydroxyl regions and positive humps in the 1800–2800 cm^−1^ region, indicating the adsorption of formaldehyde and changes of background due to free charge carrier adsorption through oxygen chemisorption. This suggested that at RT, only the SnO_2_/TiO_2_@Pt nanocomposite can catalyze the oxidative decomposition of HCHO molecules.

According to the obtained results of DRIFTS analysis, we can conclude that surface formate and DOM species are the main intermediates for HCHO oxidation, and propose the following mechanism for the low-temperature detection of HCHO by the obtained nanocomposites. Being soft based, formaldehyde is mainly adsorbed on Bronsted acid sites—hydroxyl groups via H-bonding interaction (Equation (2)) [10,57]. This follows from the monotonic decrease in the intensity of OH group vibrations and an increase in the intensity of formate vibrations with the time of formaldehyde exposure (Figure 10a). Since the number of surface OH groups for all samples is approximately the same (Figure S6, Supplementary Materials), the number of adsorbed HCHO molecules at the first step will also be approximately the same. At the next stage, adsorbed formaldehyde molecules oxidize by chemisorbed oxygen ions and produce DOM intermediate (Equation (3)) and then formate species (Equation (4)). Further, the enhancement of the signal to HCHO will be dictated by the catalytic activity of the sample. In particular, Pt and Au nanoparticles can accelerate the oxidation process due to their high catalytic activity. Finally, during the last process, these intermediates could be completely oxidized into CO_2_ and H_2_O (Equation (5)).

(2) $$HCHO_{\left(gas\right)}\to HCHO_{\left(ads\right)}$$

(3) $$\beta \cdot HCHO_{\left(ads\right)}+O_{\beta \left(ads\right)}^{\alpha -}\to \beta \cdot H_{2}COO_{\left(ads\right)}^{-}+\left(\alpha -1\right)\cdot e^{-}$$

(4) $$\beta \cdot H_{2}COO_{\left(ads\right)}^{-}+O_{\beta \left(ads\right)}^{\alpha -}\to \beta \cdot HCOO_{\left(ads\right)}^{2-}+\beta \cdot OH_{\left(ads\right)}^{\left(\alpha -1\right)-}$$

(5) $$2\beta \cdot HCOO_{\left(ads\right)}^{2-}+O_{\beta \left(ads\right)}^{\alpha -}\to 2\beta \cdot CO_{2}+\beta \cdot H_{2}O+\left(4\beta +\alpha \right)\cdot e^{-}$$

As was mentioned above, the formation of *n-n* heterocontact at the SnO_2_/TiO_2_ interface can lead to electron transfer from the TiO_2_ conduction band (E_c_) into the SnO_2_ conduction band since E_c_ (TiO_2_-anatase) > E_c_ (SnO_2_). This, consequently, can lead to an increase in the amount of chemisorbed oxygen, as was shown by the O1s XP spectrum (Figure 4). The effect of gold and platinum nanoparticles on the enhancement of the sensor signal of composites can be described by the chemical and electronic sensitization mechanisms [25,32]. Au NPs are located on the SnO_2_/TiO_2_ surface in metallic form, therefore the chemical mechanism of interaction with the gas phase is more typical. The mechanism of chemical sensitization in this case can proceed as follows. Oxygen molecules undergo dissociative adsorption on the surface of Au NPs due to the lower required activation energy. Therefore, the oxidation of formaldehyde molecules occurs more actively by atomic oxygen forms O^-^, which migrate to the surface of the semiconductor grains. As a result of this process, electrons are released into the conduction band of the semiconductor metal oxide, providing an increased sensor response [58,59].

Despite the fact that the synthesis annealing temperature of the nanocomposites was gentle, it can be observed that the platinum nanoparticles, due to their small size, are oxidized to PtO. However, during sensor measurements when the samples were heated in an atmosphere of a reducing gas, PtO can be reduced to Pt^0^. Based on the TPR-H_2_ profile of an SnO_2_/TiO_2_@Pt sample (Figure 6) this can most likely happen at 130 °C. As reported by Ono and coworkers [60], the thermal stability of Pt oxides is the lowest for Pt NPs supported on TiO_2_; regardless of the annealing environment (UHV or O_2_), the maximum content of the Pt^0^ component was observed when Pt/TiO_2_ was annealed at 500 K. In this way, the influence of the chemical interaction mechanism can be predominant at higher temperatures, leading to oxygen spill-over with further oxidation of HCHO molecules. At the same time, when Pt NPs are in the oxidized form of PtO at lower temperatures, the electronic sensitization mechanism can occur. The work function of the platinum in the oxidized state (6.8 eV) is more than that in the metallic state (5.65 eV) [61,62]. In this case, PtO acts as an electron acceptor and the increased difference in work function comparing with SnO_2_ (4.9 eV) and TiO_2_ (4.2 eV) produces an electron-depleted space-charge layer at the interface with semiconductor support [10,63,64]. This was verified by increasing resistance (Figure 7a) and positive shift of the Ti2p XP-spectrum by 0.4 eV (Figure 5a). HCHO molecules can directly interact with PtO, undergoing oxidation and leading to a change in the oxidation state of PtO.

## 4. Conclusions

SnO_2_/TiO_2_ nanocomposites were synthesized by a chemical precipitation method and then decorated with Au or Pt nanoparticles. According to TEM analysis and XRD patterns, SnO_2_ crystallites have a near spherical shape with 4 ± 1 nm size in diameter. Pt NPs and TiO_2_ are well dispersed on the SnO_2_ surface, while Au NPs are located in the form of individual spherical particles with a size of 17 ± 4 nm.

The results of sensor measurements showed that the modification of SnO_2_ with TiO_2_ allows the reduction of the temperature of HCHO detection from 150 to 100 °C. Modification of SnO_2_/TiO_2_ nanocomposites with Au NPs increases the sensor signal at T = 100 °C, while modification with Pt NPs provides the sensor signal at T = 25 °C and 50 °C. Furthermore, it was shown that the obtained nanocomposites exhibit high selective sensitivity in formaldehyde detection within the sub-ppm concentration range among different VOCs.

The influence of the TiO_2_ phase on the enhancement of sensor response toward HCHO and other VOCs is based on the formation of *n-n* heterocontact at the SnO_2_/TiO_2_ interface, leading electron transfer from E_c_ (TiO_2_) into E_c_ (SnO_2_) and consequently increasing the amount of chemisorbed oxygen, which was shown by the O1s XPS spectrum and shifting of the TPR-H_2_ peak in the low-temperature region.

The sensitizing effect of Au NPs and Pt NPs has a different origin. The presence of noble metal NPs on the surface of metal oxides can reduce potential barriers of the nucleophilic O_2_^-^ interaction with the analyte-gas molecule due to their high catalytic activity. Thus, for SnO_2_/TiO_2_@Pt samples, the mechanism of electronic sensitization is characteristic at low operating temperatures, while with an increase in the measurement temperature in reducing atmospheres, PtO is reduced to Pt and, therefore, the mechanism of chemical sensitization will prevail. It was additionally proven by the appearance of formate and DOM intermediates only in the SnO_2_/TiO_2_@Pt DRIFT spectrum at room temperature, indicating oxidative decomposition of HCHO molecules, while at 100 °C the same intermediates appeared for all samples.

For the SnO_2_/TiO_2_@Au sample, a spill-over effect of oxygen is responsible for the increase in the sensor response in the entire temperature range.

## Figures and Tables

**Table 1:** Microstructure characteristics and composition of investigated samples.

Sample	d_XRD_ (SnO_2_), nm	d_TEM_ (SnO_2_), nm	[M]*/([Sn]+[Ti]+[M]*), Mass. %	[Ti]/([Ti]+[Sn]+[M]*), mass. %	S_surf_, m^2^/g
SnO_2_	4 ± 1	4.5 ± 1	-	-	115 ± 5
SnO_2_/TiO_2_			-	1.4 ± 0.1	97 ± 4
SnO_2_/TiO_2_@Pt			1.0 ± 0.1	1.2 ± 0.1	90 ± 4
SnO_2_/TiO_2_@Au			1.2 ± 0.1	1.3 ± 0.1	85 ± 4

Notes; **d_XRD_**—crystallite size from XRD; **d_TEM_**—particle size from TEM; [M]*—Pt and Au, respectively; **S_surf_**—specific surface area.

**Table 2:** Minimum detectable HCHO concentration ***c_min_*** measured at 200 °C for sensors based on investigated materials.

Sample	SnO_2_	SnO_2_/TiO_2_	SnO_2_/TiO_2_@Pt	SnO_2_/TiO_2_@Au
***c_min_*, pp** **b**	72	33	111	21

## Data Availability

The data presented in this study are available on request from the corresponding author. The data are not publicly available due to privacy reason.

## Supplementary Materials

The following are available online at https://www.mdpi.com/article/10.3390/nano11082049/s1, Figure S1: Schematic illustration of the synthesis of the SnO_2_/TiO_2_ nanocomposites modified with Au NPs and Pt NPs., Figure S2: XRD pattern (a) and Raman spectrum (b) of TiO_2_-300, Figure S3: TEM image and size distribution of the AuNPs determined by TEM images analysis (inset) (a), XRD pattern (b) and absorption spectrum (c) of synthesized AuNPs, Figure S4: TEM image and size distribution of the SnO_2_ nanoparticles determined by TEM image analysis (inset), Figure S5: Sn 3d XP-spectra of the synthesized samples, Figure S6: FTIR spectra of the synthesized samples.
