# Supplementary material for: Low Temperature HCHO Detection by SnO2/TiO2@Au and SnO2/TiO2@Pt: Understanding by In-Situ DRIFT Spectroscopy
Source: Nanomaterials (Basel). 2021 Aug 11;11(8):2049. doi: 10.3390/nano11082049 (PMC8398349; doi:10.3390/nano11082049)
Supplement: Supplementary file 1 [file nanomaterials-11-02049-s001.zip › nanomaterials-1314184-supplementary material xml.pdf]

nanomaterials

MDPI

Supplementary Material

Low temperature HCHO detection by SnO_2/TiO_2@Au and SnO_2/TiO_2@Pt: understanding by in-situ DRIFT spectroscopy

Abulkosim Nasriddinov [1,2], Vadim Platonov [1], Alexey Garshev [1,2] and Marina Rumyantseva [1,*]

[1] Department of Chemistry, Lomonosov Moscow State University, Leninskie Gory 1-3, Moscow, 119991, Russia; a.f.nasriddinov@gmail.com (A.N.); agnes1992@yandex.ru (V.P.); gaal@mail.ru (A.G.)

[2] Department of Materials Science, Lomonosov Moscow State University, Leninskie Gory 1-3, Moscow, 119991, Russia

* Correspondence: roum@inorg.chem.msu.ru; Tel.: +7-495-939-5471

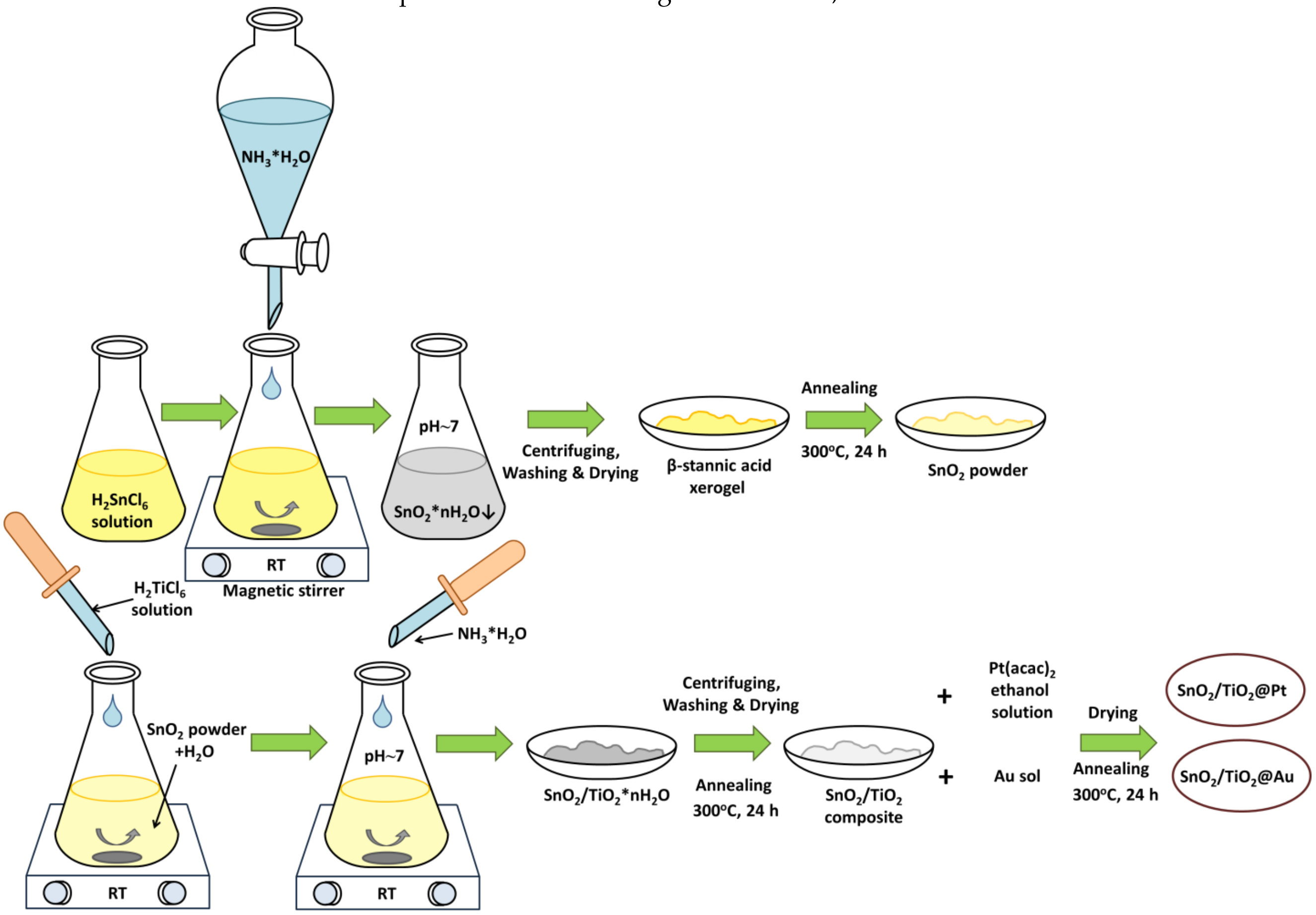

Figure S1. Schematic illustration of the synthesis of the SnO_2/TiO_2 nanocomposites modified with Au NPs and Pt NPs.

Nanocrystalline TiO_2 was obtained under similar synthesis conditions and was annealed at 300 °C for 24 hours in air. According to the ICDD PDF-2 database, all peaks on the XRD pattern (Figure S2a) correspond to the anatase phase. Raman peaks observed at 137.5, 194.6, 392, 513.5 and 632.7 cm^{-1} are attributed to E_g, E_g, B_{1g}, A_{1g}+B_{1g} and E_g modes of the anatase phase (Figure S2b), respectively [1]. The obtained results indicate that such synthesis conditions can lead to formation of single-phase of TiO_2 sample.

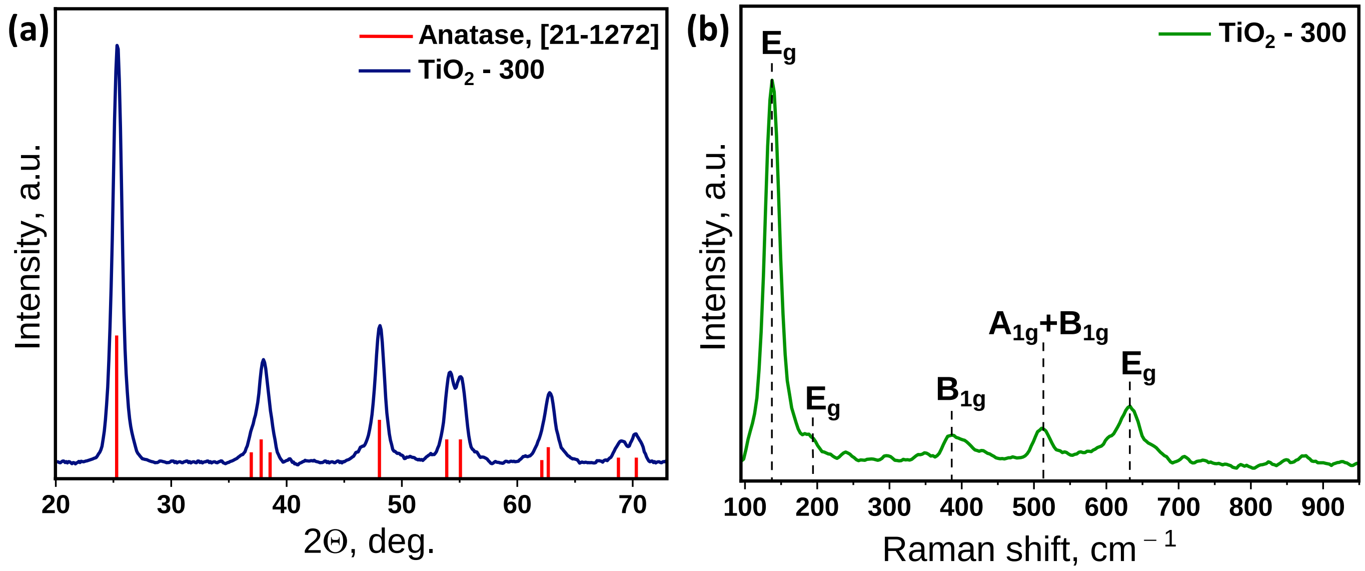

Figure S2. XRD pattern (a) and Raman spectrum (b) of TiO_2-300.

TEM examination of Au sol showed the spherical shape gold nanoparticles (Au NPs) with an average particle size of 17 ± 4 nm (Figure S3a). XRD pattern showed the presense of cubic gold phase (space group Fm3m, Figure S3b). Calculated crystallite size by Sherrer equation (16 ± 2 nm) is equal as that obtained by the TEM method. Due to the strong absorption in the green region of the spectrum (λ_{max} = 520 nm), which is assosiated with localized surface plasmon resonance (LSPR) effect, Au sol is ruby red in color (Figure S3c). Symmetric LSPR band indicates a narrow size distribution of nanoparticles.

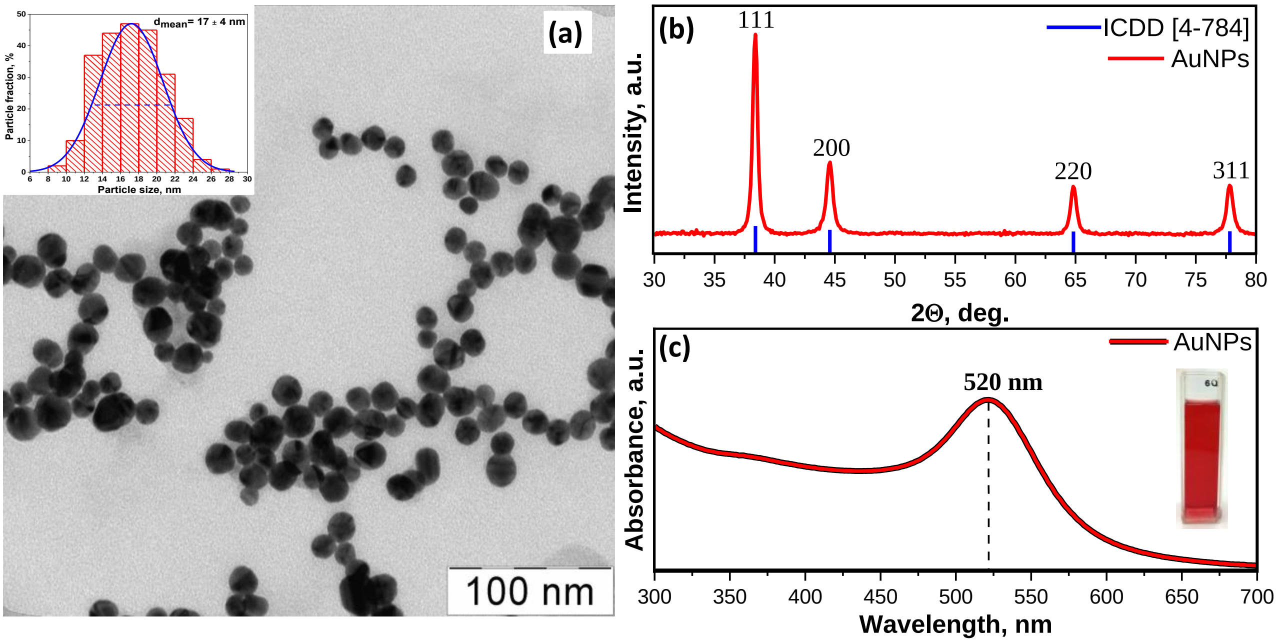

Figure S3. TEM image and size distribution of the AuNPs determined by TEM images analysis (inset) (**a**), XRD pattern (**b**) and absorption spectrum (**c**) of synthesized AuNPs.

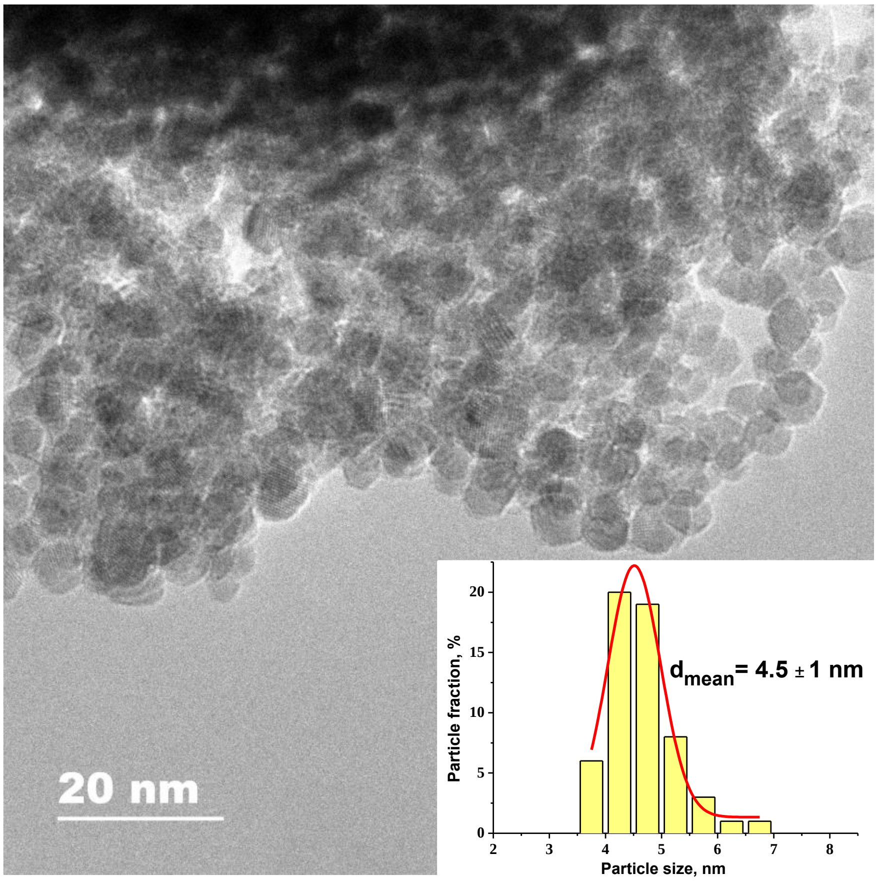

Figure S4. TEM image and size distribution of the SnO_2 nanoparticles determined by TEM image analysis (inset).

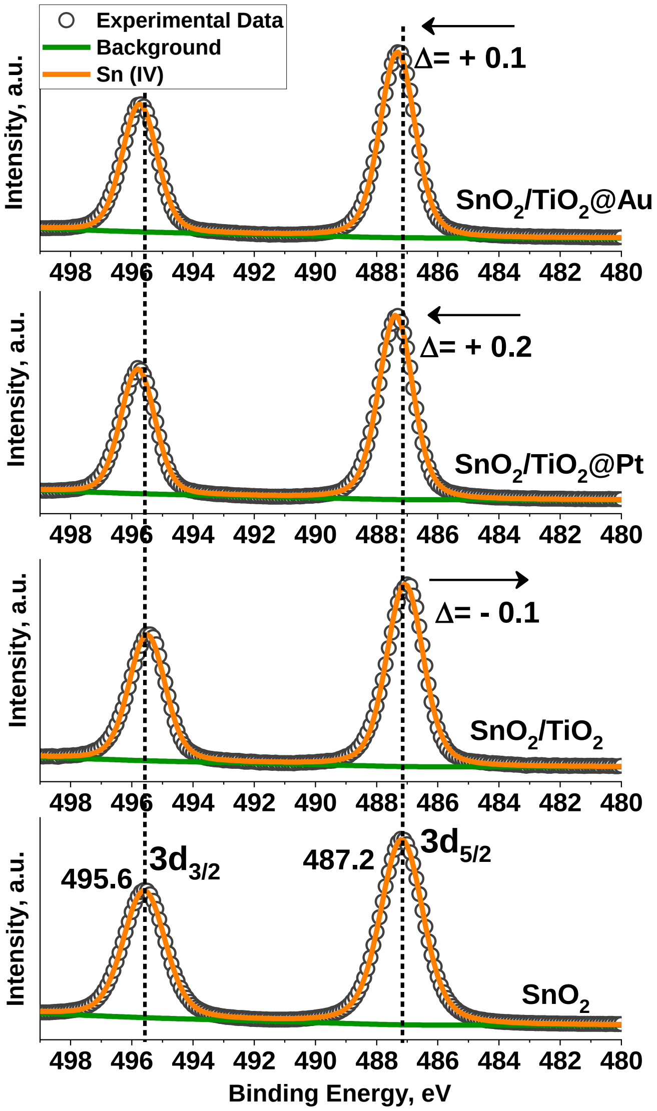

Figure S5. Sn 3d XP-spectra of the synthesized samples.

FTIR spectra of nanocomposites (Figure S6) consist of intense and wide band associated to Sn-O (530 cm^{-1}) and O-Sn-O (620 cm^{-1}) vibration modes [2]. A peak at 1628 cm^{-1} is attributed to deformation vibrations of water molecules and a broad peak at the 2500 – 3700 cm^{-1} wavelength range is associated with the stretching vibrations of the surface OH groups [3]. According to the results of FTIR spectroscopy, it can be seen that the introduction of modifiers does not affect the surface OH groups. Other possible vibration modes from modifiers did not observed due to their low concentration in the composition of nanocomposites.

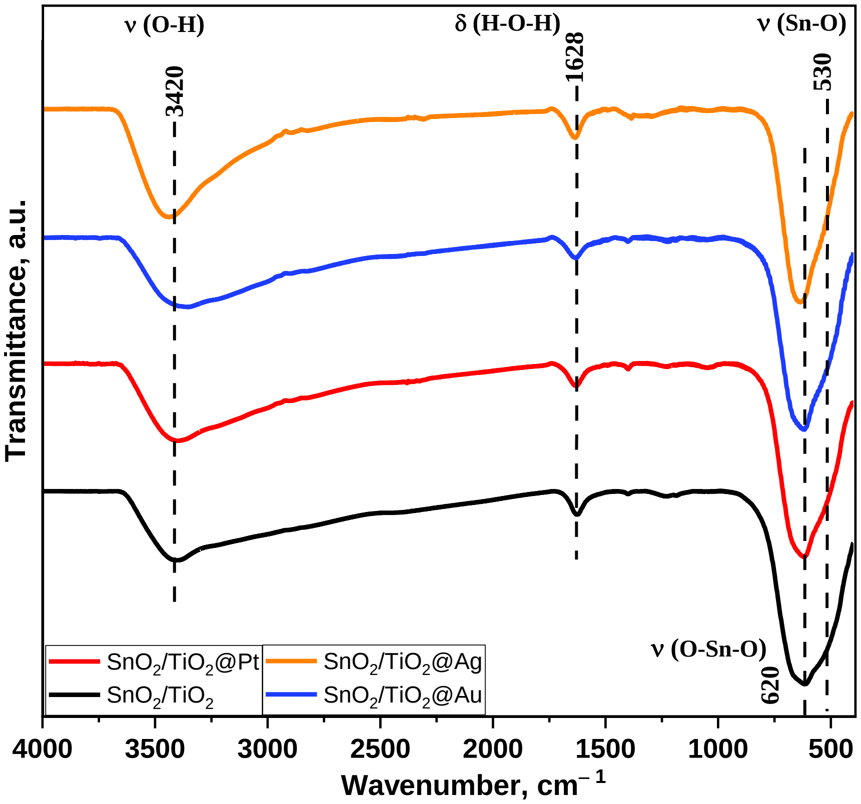

Figure S6. FTIR spectra of the synthesized samples.

References

1. Loudon, R. The Raman effect in crystals. *Adv. Phys*. **1964**, *13*, 423–482.
2. Nasriddinov, A.; Rumyantseva, M.; Marikutsa, A.; Gaskov, A.; Lee, J.H.; Kim, J.H.; Kim, J.Y.; Kim, S.S.; Kim, H.W. Sub-ppm Formaldehyde Detection by n-n $TiO_2@SnO_2$ Nanocomposites. *Sensors*, **2019**, *19*, 3182.
3. Nakamoto, K. Infrared and Raman Spectra of Inorganic and Coordination Compounds. Part A: Theory and Applications in Inorganic Chemistry, 6th ed.; Publisher: John Wiley & Sons, Inc.: Hoboken, NJ, USA, 2009.
